# Developing a Decision Aid for Clinical Obesity Services in the Real World: the DACOS Nationwide Pilot Study

**DOI:** 10.1007/s11695-024-07123-6

**Published:** 2024-03-11

**Authors:** Evan Atlantis, Nic Kormas, Milan Piya, Mehdi Sahebol-Amri, Kathryn Williams, Hsin-Chia Carol Huang, Ramy Bishay, Viral Chikani, Teresa Girolamo, Ante Prodan, Paul Fahey

**Affiliations:** 1 School of Health Sciences, Western Sydney University, Campbelltown Campus, Locked Bag 1797, Penrith, NSW Australia; 2 Department of Endocrinology, Concord Hospital, Concord, New South Wales Australia; 3 South Western Sydney Metabolic Rehabilitation and Bariatric Program, Camden and Campbelltown Hospitals, Campbelltown, New South Wales Australia; 4 School of Medicine, Western Sydney University, Campbelltown, New South Wales Australia; 5 Ryde Hospital, Northern Sydney Local Health District, Ryde, New South Wales Australia; 6 Department of Endocrinology, Nepean Hospital, Nepean Blue Mountains Local Health District, Kingswood, New South Wales Australia; 7 Charles Perkins Centre-Nepean, The University of Sydney, Kingswood, New South Wales Australia; 8 Respiratory & Sleep Medicine, Canberra Hospital, Garran, Canberra, Australian Capital Territory Australia; 9 Canberra Obesity Management Service, Canberra Health Services, Belconnen, Canberra, Australian Capital Territory Australia; 10 College of Health and Medicine, Australian National University, Acton, Australian Capital Territory Australia; 11 Metabolic & Weight Loss Clinic, University Clinics, Western Sydney University, Blacktown Hospital, Blacktown, New South Wales Australia; 12 Department of Diabetes and Endocrinology, Princess Alexandra Hospital, Brisbane, Queensland Australia; 13 Re:You Health, Adelaide Weight Management and Wellness, Adelaide, South Australia Australia; 14 School of Computer, Data and Mathematical Sciences, Western Sydney University, Sydney, Australia

**Keywords:** Management, Obesity, Weight loss, Decision support model

## Abstract

**Purpose:**

The purpose of this study is to develop a decision aid tool using “real-world” data within the Australian health system to predict weight loss after bariatric surgery and non-surgical care.

**Materials and Methods:**

We analyzed patient record data (aged 16+years) from initial review between 2015 and 2020 with 6-month (*n*=219) and 9-/12-month (*n*=153) follow-ups at eight clinical obesity services. Primary outcome was percentage total weight loss (%TWL) at 6 months and 9/12 months. Predictors were selected by statistical evidence (*p*<0.20), effect size (±2%), and clinical judgment. Multiple linear regression and bariatric surgery were used to create simple predictive models. Accuracy was measured using percentage of predictions within 5% of the observed value, and sensitivity and specificity for predicting target weight loss of 5% (non-surgical care) and 15% (bariatric surgery).

**Results:**

Observed %TWL with bariatric surgery vs. non-surgical care was 19% vs. 5% at 6 months and 22% vs. 5% at 9/12 months. Predictors at 6 months with intercept (non-surgical care) of 6% include bariatric surgery (+11%), BMI>60 (–3%), depression (–2%), anxiety (–2%), and eating disorder (–2%). Accuracy, sensitivity, and specificity were 58%, 69%, and 56%. Predictors at 9/12 months with intercept of 5% include bariatric surgery (+15%), type 2 diabetes (+5%), eating disorder (+4%), fatty liver (+2%), atrial fibrillation (–4%), osteoarthritis (–3%), sleep/mental disorders (–2–3%), and ≥10 alcohol drinks/week (–2%). Accuracy, sensitivity, and specificity were 55%, 86%, and 53%.

**Conclusion:**

Clinicians may use DACOS to discuss potential weight loss predictors with patients after surgery or non-surgical care.

**Graphical Abstract:**

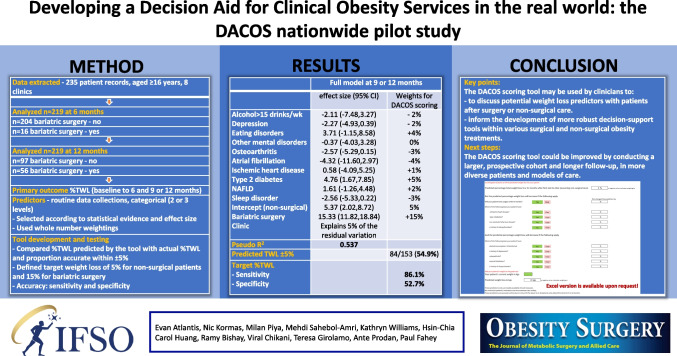

**Supplementary Information:**

The online version contains supplementary material available at 10.1007/s11695-024-07123-6.

## Introduction

Obesity is a global epidemic, with rates nearly tripling in most countries since 1975 [1]. The rising health problems associated with obesity are undoubtedly challenging health systems worldwide [2]. In Australia, approximately 70% of adults with overweight or obesity (7 million people) likely have weight-related complications, including medical, psychological, and functional impairments [3]. These complications increase with obesity severity, are associated with avoidable health service utilization, and should be addressed as soon as possible [4].

As the first point of contact for most people seeking healthcare services, general practice remains at the forefront of efforts to prevent and manage obesity [2]. Although there are comprehensive, evidence-based guidelines on how to provide effective weight management in general practice [5], obesity and related complications remain undiagnosed and undertreated [6–8]. With rising rates of more severe forms of obesity, patients often present with multiple health conditions and, consequently, complex health needs that cannot be met within the constraints of most primary care settings. Their health needs may be more appropriately addressed in public hospitals or private clinics that specialize in obesity assessment and management [9]. These services, known as “clinical obesity services,” usually require a referral from a patient’s general practitioner. They can provide more intensive treatments and care, including bariatric surgery and non-surgical multidisciplinary care.

Clinical obesity services have been shown to effectively improve a range of health care and health outcomes in patients with severe obesity [10–13]. However, it is important to note that not everyone who is referred will be successful. By understanding some factors that can predict weight loss [14], people with severe obesity can increase their chances of success. This information and patient preferences can guide evidence-based treatment pathways for weight loss and related clinical outcomes over time. This is needed to support advanced (shared and informed) decision-making on the most appropriate treatments and set realistic treatment expectations. To address this issue, we aimed to evaluate outcomes in clinical obesity services and develop a decision aid tool using “real world” data within the Australian healthcare system.

## Materials and Methods

### Study Design and Population

The DACOS was a nationwide case series study with a pre-test and post-test design. We collected de-identified data extracted from electronic and/or paper-based medical records at eight clinics, including private and public hospital clinics. We identified patients with obesity who had a body mass index (BMI) of 30 kg/m^2^ or higher, referred for clinical obesity services, and followed for at least 6 months and up to 24 months, from their initial review between 2015 and 2020. There were no specific exclusion criteria for patients identified as eligible. To date, data from 273 patients across eight clinics in Australia have been extracted. For this study, we selected records for patients aged 16 years and over for whom weight loss could be estimated at 6 and 9 or 12 months after first clinic visit.

### Patient Data

The data extracted from patients for this study included demographic information, anthropometric measurements, medical history, comorbidities, procedures, and current medications as part of routine clinical practice. The data also included information on services provided, such as the use of continuous positive airway pressure devices, the use of diet replacement products, the use of pharmacotherapies for weight management, and whether bariatric surgery was received.

### Outcome Variable

The primary outcome was percentage total weight loss (%TWL) from baseline to 6 months and 9 or 12 months. Data were recorded at baseline and then at 3-month intervals to 24 months. Although few patients visited the clinic at such regularly spaced times, we sought to extract data from the first patient visit within one month of the target date. Patients’ current weight was missing whenever no visit was made during that time period or a visit was made but patient weight was not recorded. Where weight was missing for one or more visits, but available from both an earlier and later time point, we used linear interpolation to impute the missing weights. When the patient’s final weight measurement was at 9 months, we included their weight at 9 months in the 12-month analysis. Summary statistics are also provided for %TWL, change in BMI, and the proportion of patients achieving weight loss targets (defined as at least 5% of total body weight for non-surgical patients and at least 15% of total body weight for those who received surgical interventions).

### Predictors of %TWL

We considered measurements which would typically be available to general practitioners when discussing referral to weight management clinics with their patients. These included demographics (age, sex), body mass index, smoking and alcohol status, mental and physical health history, number of medications, and current use of a continuous positive airway pressure (CPAP) machine. To keep the scoring system as simple as possible while incorporating the most important information, predictors were expressed as categorical variables with just two or three categories.

### Tool Development and Testing

We aimed to develop a tool which could aid decision planning around referral to an obesity care clinic using information readily accessible to patients and their general practitioners. Predictors were selected according to statistical evidence, effect size, and clinical judgment. For simplicity, we only used whole number weightings.

The most important test of the decision aid was whether it could accurately predict future weight loss. We compared the %TWL predicted by the tool with actual %TWL recorded by the individuals in our data set and report the proportion of predictions that were accurate within ±5%. We also defined a target weight loss of 5% for non-surgical patients and 15% for those undergoing bariatric surgery and checked how well the tool could predict the achievement of these targets. Accuracy is reported in terms of sensitivity and specificity.

### Statistical Methods

To ensure compatibility across clinics, we developed standard definitions of variables (data dictionary) and converted all incoming data into a single common format. We checked the distribution of each variable using frequency counts (for categorical variables) or histograms (for numeric variables) and checked the relationships between pairs of variables using cross-tabulations, side-by-side boxplots, and scatterplots as appropriate. We referred any questions to the data provider (clinical lead at each site) for verification.

As the participants were clustered by clinic, participants within the same clinic may be more similar and have more similar results than participants from different clinics. We visually inspected for outlier clinics using side-by-side boxplots and tested for outlier clinics by fitting clinic as a fixed effect in the regression models. As bariatric surgery is a strong predictor of weight loss and its availability differed between clinics, the confounding effect of bariatric surgery was adjusted for by including it in the model.

Even after adjustment for the use of bariatric surgery, variations in %TWL were observed between clinics. Therefore, all regression models include clinic as a random effect, allowing appropriate adjustment for clinic-based clustering when reporting evidence of association between the predictor variables and the outcome. We modeled clinic as a random effect rather than a fixed effect as fixed effects would compromise the confidentiality of participating clinics and undermine the generalizability of the resulting tool.

For each potential predictor, a mixed effect linear regression model of %TWL adjusted for the use of bariatric surgery (fixed effect) and clinic (random effect), was fit. These models provide estimates of both clinical effect size (the regression coefficient and 95% confidence intervals) and statistical evidence of association (as *p*-values). Pseudo *R*^2^ [15] statistics are also reported.

Review of the standardized residuals and Cook’s distances for each individual in the data set confirmed the presence of high and low outliers and some influential observations. Weight loss can be highly variable between individuals and challenging to accurately model. One outlier, for example, was an individual who lost 1/3 of their body weight from baseline without surgery, while another individual managed to gain a little weight despite bariatric surgery. Although outliers, there is no reason to doubt the validity of these data. We used 95% winsorization to limit the influence of extreme observations during model fitting, but all model testing was conducted on the original observations.

Candidate predictors which displayed a clinical meaningful effect (±2% weight loss), any statistical evidence of association (*p*<0.20) or which were deemed to be clinically meaningful predictors were combined in a single multivariate mixed model that provides the basis for the decision aid tool.

To produce the decision aid tool, we rounded the regression coefficients of the full model to the closest integer. The success of the prediction is reported as the proportion of predictions falling within 5 percentage points of actual %TWL and the sensitivity and specificity of the prediction in meeting weight loss targets.

### Sample Size Estimation per Site, Cluster Adjusted

As there was no specific hypothesis testing, choice of sample size was somewhat subjective. Clearly the larger the sample size, the better we would be able to specify the predictive model. A minimum sample target of 30 participants (each with complete data) from each of eight sites would have at least 80% power to detect a 0.25 standard deviation decrease in mean %TWL (from baseline to follow-up) assuming the intra-class correlation is 0.05 or less. (GLIMMPSE online calculator https://glimmpse.samplesizeshop.org).

### Supplementary Analyses

As an external validation, we compared the %TWL predicted by our DACOS tool with the %TWL predictions from the American College of Surgeons Metabolic and Bariatric Surgery Accreditation and Quality Improvement Program (MBSAQIP) surgical risk/benefit calculator (Supplementary 2).

## Results

A review of eligible patient records found that 38 out of 273 patient records across eight clinic study sites were excluded for being aged less than 16 years (Supplementary Figure 1). After excluding those without a weight recording, there were 219 records available for analysis at 6 months and 153 records available for analysis at 9 or 12 months. Baseline characteristics of patients originally selected, patients included in the analyses at 6 months, and those included in the analysis at 9 or 12 months appear below (Table 1). Those with a history of eating disorders had a high loss to follow-up, decreasing from 13% of the sample at baseline to 8% of the remaining sample at 9 or 12 months. Conversely, those with type 2 diabetes had a lower loss to follow-up, increasing from 50% at baseline to 56% of the remaining sample at 9 or 12 months.

**Table 1:** Baseline characteristics of study participants

	Total at baseline	Total at 6 months	Total at 9 or 12 months
	*n*=235	*n*=219	*n*=153
Gender			
Female, *n* (%)	153 (65%)	143 (65%)	100 (65%)
Age in years,			
Mean (95% CI)	46 (44,48)	47 (45,49)	47 (44,49)
Age			
16–19 years	16 (7%)	15 (7%)	13 (9%)
20–29 years	23 (10%)	21 (10%)	13 (9%)
30–39 years	38 (16%)	33 (15%)	21 (14%)
40–49 years	48 (20%)	46 (21%)	29 (19%)
50–59 years	57 (24%)	55 (25%)	40 (26%)
60–69 years	44 (19%)	41 (19%)	30 (20%)
70–79 years	9 (4%)	8 (4%)	7 (6%)
Country of birth			
Australia	153 (74%)	140 (74%)	101 (74%)
NZ, UK, Ireland, Canada, USA	22 (11%)	14 (10%)	14 (10%)
Elsewhere	31 (15%)	21 (15%)	21 (15%)
Missing	29	29	15
Aboriginal or Torres Strait Islander			
Yes, *n* (%)	11 (6%)	10 (6%)	8 (6%)
Missing	60	59	24
Married/de facto			
*n* (%)	118 (51%)	111 (51%)	76 (51%)
Missing	3	3	3
Employed			
*n* (%)	93 (41%)	85 (40%)	55 (37%)
Missing	7	7	4
Baseline weight kg,			
Mean (95% CI)	138 (134,142)	138 (133,142)	138 (133,143)
Missing	1	0	0
Baseline BMI			
Mean (95% CI)	49 (47,50)	49 (47,50)	49 (47,51)
Missing	12	11	7
Alcohol risk			
Low (<10 dkr/wk)	218 (94%)	202 (94%)	144 (94%)
Medium (10–15 drk/wk)	6 (3%)	6 (3%)	5 (3%)
High (>15 drk/wk)	7 (3%)	7 (3%)	4 (3%)
Missing	4	4	0
Smoking status			
Never	123 (54%)	112 (53%)	74 (49%)
Former	75 (33%)	73 (34%)	55 (36%)
Current	31 (13%)	28 (13%)	22 (15%)
Missing	6	6	2
Nocturnal ventilation			
Some history at baseline	77 (33%)	70 (32%)	48 (31%)
Number of medications			
0–3 at baseline	91 (39%)	83 (38%)	62 (41%)
4–7 at baseline	71 (31%)	66 (30%)	38 (25%)
>7 at baseline	70 (30%)	68 (31%)	51 (34%)
Missing	3	2	0
Diagnoses at or before baseline			
Depression	112 (48%)	107 (49%)	71 (46%)
Anxiety	49 (21%)	44 (20%)	29 (19%)
Eating disorders	30 (13%)	28 (13%)	12 (8%)
Other mental health disorder	38 (16%)	36 (16%)	24 (16%)
Osteoarthritis	88 (37%)	84 (38%)	63 (41%)
Autoimmune disease	25 (11%)	23 (11%)	15 (10%)
Gout	7 (3%)	7 (3%)	5 (3%)
Chronic back pain	55 (23%)	49 (22%)	32 (21%)
Other musculoskeletal conditions	30 (13%)	30 (14%)	19 (12%)
Arterial fibrillation	11 (5%)	10 (5%)	5 (3%)
Heart failure	15 (6%)	14 (6%)	8 (5%)
Ischemic heart disease	19 (8%)	19 (9%)	14 (9%)
Hypertension	132 (56%)	127 (58%)	89 (58%)
Other heart disorders	18 (8%)	17 (8%)	11 (7%)
Peripheral vascular disorder	23 (10%)	21 (10%)	17 (11%)
Stroke	8 (3%)	5 (2%)	1 (<1%)
Type 1 diabetes	2 (<1%)	2 (1%)	0
Type 2 diabetes	117 (50%)	112 (51%)	85 (56%)
Chronic kidney disease stage 2-5	18 (8%)	18 (8%)	13 (9%)
Non-alcoholic fatty liver	72 (31%)	66 (30%)	49 (32%)
Reflux	62 (26%)	61 (28%)	44 (29%)
Gallstones	10 (4%)	9 (4%)	4 (3%)
Gastrointestinal disorders	2 (<1%)	2 (1%)	0
Sleep disorders	105 (45%)	96 (44%)	70 (46%)

Weight loss outcomes were significantly greater among those who received bariatric surgery compared with non-surgical patients at both 6 months and 9 or 12 months (Table 2), and surgery rates at 9 to 12 months varied from 0 to 75.9% between clinics (0 to 41.4% at 6 months), making the use of surgery a strong confounder of the %TWL outcome.

**Table 2:** Summary measures of weight loss for bariatric surgery and non-surgical care groups at 6 months and 9 or 12 months.

	Non-surgical care	Bariatric surgery	Difference between groups	
			change (95% CI)	*p*-value
Weight loss at 6 months	*n*=204	*n*=15		
Kilograms lost (mean)	6.0 kg	22.9 kg	−17.0 (−23.9, −10.1)	<0.001
%TWL (mean)	4.6 %	9.4 %	−14.8 (−20.6, −9.0)	<0.001
BMI (mean)^a^	2.2 kg/m^2^	8.1 kg/m^2^	−5.9 (−8.3, −3.4)	<0.001
Achieved target weight loss (%)	87 (43%)	10 (67%)		
Weight loss at 9 or 12 months	*n*=97	*n*=56		
Kilograms (mean)	6.9 kg	28.3 kg	−21.3 (−25.6, −17.0)	<0.001
%TWL (mean)	4.9 %	21.7 %	−16.7 (−19.7, 13.8)	<0.001
BMI (mean)^b^	2.3 kg/m^2^	10.1 kg/m^2^	−7.8 (−9.5,−6.0)	<0.001
Achieved target weight loss (%)	36 (37%)	43 (77%)		

^a^BMI at 6 months missing for 9 patients in the no surgery group

^b^BMI at 9 or 12 months missing for 12 patients in the non-surgical care group and 12 patients in the bariatric surgery group

Each potential predictor was checked for evidence of association with %TWL after adjustment for use of bariatric surgery and variation between clinics. Summary results are presented below (Table 3). Highlighted predictors (bold) displayed either a >2% effect size (coefficient) or *p*<0.2 significance level and were included in the predictive model.

**Table 3:** Relationship between each predictor and %TWL after adjusting for clinic and use of bariatric surgery

	At 6 months		At 9 or 12 months	
	effect size (95% CI)	*p*-value	Coefficient (95% CI)	*p*-value
Gender				
Female	−0.44 (−2.16, 1.29)	0.618	−1.20 (−3.92, 1.53)	0.387
Age		0.537		0.226
16–19 years	−0.99 (−6.74, 4.75)	0.729	0.15 (−8.07, 8.37)	0.968
20–29 years	−2.45 (−5.56, 0.65)	0.121	5.65 (0.44, 10.85)	0.034
30–39 years	−0.49 (−3.17, 2.19)	0.720	0.66 (−3.69, 5.00)	0.765
40–49 years	−0.50 (−2.89, 1.88)	0.679	−0.69 (−4.53, 3.14)	0.721
50–59 years	Reference		Reference	
60–69 years	1.17 (−1.31, 3.65)	0.352	2.66 (−1.21, 6.52)	0.176
70–79 years	−0.17 (−4.78, 4.45)	0.944	−2.04 (−8.61, 4.53)	0.540
Age				
16–24 years	−2.46 (−5.96, 1.04)	0.226	0.73 (−4.65, 6.12)	0.961
25–49 years	−1.17 (−2.92, 0.58)	0.166	0.05 (−2.81, 2.91)	0.783
50 or more years	Reference	0.189	Reference	0.972
Baseline BMI >60 kg/m^2^	**−2.48** (−5.09, 0.12)	**0.062**	−1.49 (−6.06, 3.08)	0.519
Alcohol >15 drinks/wk	**−1.99**^**a**^ (−5.46, 1.47)	0.258	**−2.08** (−7.56, 3.40)	0.454
Current smoker	−1.22 (−3.73, 1.29)	0.340	−0.96 (−4.87, 2.94)	0.627
Nocturnal ventilation	1.22 (−0.54, 2.98)	**0.174**	−1.60 (−4.40, 1.21)	0.262
Number of medications				0.358
0–3 at baseline	−0.10 (−2.25, 2.06)	0.397	−0.04 (−3.31, 3.22)	0.980
4–7 at baseline	−1.30 (−3.38, 0.78)	0.931	2.26 (−1.22, 5.73)	0.201
>7 at baseline	Reference	0.231	Reference	
Diagnoses at or before baseline				
Depression	**−2.55** (−4.14, −0.97)	**0.002**	**−2.08** (−4.69, 0.54)	**0.119**
Anxiety	**−2.37** (−4.43, −0.30)	**0.025**	−1.82 (−5.22, 1.57)	0.290
Eating disorders	**−2.88** (−5.42, −0.35)	**0.026**	**3.78** (−1.13, 8.69)	**0.130**
Other mental health disorder	−0.95 (−3.14, 1.23)	0.392	**−2.03** (−5.61, 1.54)	0.263
Osteoarthritis	−0.04 (−1.76, 1.68)	0.961	**−2.57** (−5.22, 0.08)	**0.057**
Autoimmune disease	−0.26 (−3.03, 2.52)	0.854	−1.62 (−6.16, 2.92)	0.481
Gout	Too few		Too few	
Chronic back pain	−0.22 (−2.23, 1.79)	0.823	1.33 (−1.97, 4.64)	0.427
Other musculoskeletal conditions	−0.13 (−2.42, 2.26)	0.913	1.33 (−2.70, 5.36)	0.514
Arterial fibrillation	0.15 (−3.75, 4.04)	0.942	**−4.20** (−11.45, 3.06)	0.255
Heart failure	−0.40 (−3.69, 2.90)	0.813	0.97 (−4.83, 6.76)	0.741
Ischemic heart disease	1.87 (−0.99, 4.74)	**0.199**	**2.25** (−2.32, 6.81)	0.332
Hypertension	−0.58 (−2.32, 1.17)	0.515	0.41 (−2.37, 3.18)	0.773
Other heart disorders	1.55 (−1.50, 4.60)	0.318	1.62 (−3.39, 6.63)	0.524
Peripheral vascular disorder	0.15 (−2.63, 2.94)	0.914	1.64 (−2.51, 5.78)	0.436
Stroke	−3.1 (−8.6, 2.49)	0.914	1.53 (−14.61, 17.68)	0.851
Type 1 diabetes	1.78 (−6.71, 10.26)	0.680	Too few	
Type 2 diabetes	−1.03 (−2.90, 0.84)	0.279	**3.27** (0.29, 6.25)	**0.032**
Chronic kidney disease stage 2–5	0.29 (−2.79, 3.37)	0.853	−0.91 (−5.71, 3.89)	0.708
Non-alcoholic fatty liver	−0.67 (−2.52, 1.17)	0.473	**2.19** (−0.65, 5.03)	**0.129**
Reflux	1.03 (−0.78, 2.83)	0.264	−0.37 (−3.24, 2.50)	0.798
Gallstones	1.71 (−2.34, 5.76)	0.407	−1.18 (−9.24, 6.89)	0.773
Gastrointestinal disorders	Too few		Too few	
Sleep disorders	−0.25 (−1.91, 1.40)	0.764	**−2.34** (−4.93, 0.25)	**0.076**

^\a^Rounds off to −2.0

The fitted models and weights for the decision aid tool are summarized below (Table 4). Two models are reported: the null model and the full model containing each of the potential predictors identified (see Table 3). The model coefficients were rounded to the nearest integer to produce the weights for the decision aide tool. For each individual in the data set, the weights that apply are summed to provide a predicted weight loss. The predicted weight loss was then compared to the recorded weight loss.

**Table 4:** Fitted model and associated weights for the DACOS scoring system

	Null model at 6 months		Full model at 6 months		Null model at 9 or 12 months		Full model at 9 or 12 months	
	effect size (95% CI)	Decision aid tool	effect size (95% CI)	Decision aid tool	effect size (95% CI)	Decision aid tool	effect size (95% CI)	Decision aid tool
BMI>60 kg/m^2^			−2.60 (−5.18, −0.02)	− 3%				
Alcohol>15 drinks/wk			−1.43 (−4.88, 2.03)	− 1%			−2.11 (−7.48, 3.27)	− 2%
Nocturnal ventilation			1.16 (−0.67, 2.98)	+1%				
Depression			−1.87 (−3.65, −0.09)	− 2%			−2.27 (−4.93, 0.39)	− 2%
Anxiety			−1.65 (−4.02, 0.72)	− 2%				
Eating disorders			−2.25 (−4.97, 0.47)	− 2%			3.71 (−1.15, 8.58)	+4%
Other mental health disorders							−0.37 (−4.03, 3.28)	0%
Osteoarthritis							−2.57 (−5.29, 0.15)	−3%
Atrial fibrillation							−4.32 (−11.60, 2.97)	−4%
Ischemic heart disease			1.26 (−1.72, 4.24)	+1%			0.58 (−4.09, 5.25)	+1%
Type 2 diabetes							4.76 (1.67, 7.85)	+5%
Non-alcoholic fatty liver disease							1.61 (−1.26, 4.48)	+2%
Sleep disorder							−2.56 (−5.33, 0.22)	−3%
-Intercept	4.16 (1.10, 7.23)	4%	5.65 (2.63, 8.56)	6%	5.02 (2.11, 7.93)	5%	5.37 (2.02, 8.72)	5%
-Bariatric surgery	11.28 (7.52, 15.04)	+11%	11.15 (7.43, 14.88)	+11%	15.07 (11.58, 18.55)	+15%	15.33 (11.82, 18.84)	+15%
-Clinic	Explains 25% of the residual variation		Explains 23% of the residual variation		Explains 8% of the residual variation		Explains 5% of the residual variation	
Pseudo *R*^2^	0.147		0.216		0.437		0.537	
Predicted weight loss is within ±5%		122/219 (55.7%)		120/206 (58.3%)		71/153 (46.6%)		84/153 (54.9%)
Ability to predict achieving the weight loss target								
Sensitivity		10.3%		68.5%		100%		86.1%
Specificity		95.9%		56.1%		0%		52.7%

The full model predicted %TWL to within ±5% of the recorded value for 58.3% at 6 months and 54.9% at 9 or 12 months, improving on the null model statistics of 55.7% at 6 months and 46.6% at 9 or 12 months.

The sensitivity and specificity of the DACOS scoring system were 68.5% and 56.1% for predicting weight loss at 6 months and 86.1% and 52.7% at 9 or 12 months. This means that the DACOS correctly identified patients who did achieve target %TWL in 68.5% of patients at 6 months and 86.1% at 9 or 12 months and correctly identified patients who did not achieve target %TWL in 56.1% and 52.7% of patients at 6 months and 9 or 12 months, respectively.

### Supplementary Findings

The DACOS instrument’s predictions of %TWL at 12 months have a mean absolute error (MAE) that is on average comparable to or lower than those from the MBSAQIP instrument (Supplementary 2 Tables 2 and 3) [16]. This discrepancy may be related to the differences in baseline variables chosen by each instrument.

## Discussion

The findings of this study suggest that the DACOS scoring system may be a useful tool for clinicians in discussing potential predictors of weight loss in their patients after bariatric surgery and non-surgical care. The study found that the DACOS scoring system was able to predict the likelihood of weight loss success in patients up to 12 months after testing a combination of variables from “real world” data within the Australian healthcare system. The DACOS scoring system is easy to use, can be applied to a wide range of patients, and is also more accurate than many previous methods of predicting weight loss at 12 months [14, 16]. The predicted weight loss by the DACOS scoring system is likely to be underestimated for many patients, given that the patients referred for clinical obesity services in this study, mostly public, were heavier and more complex than those typically managed in other specialized clinical obesity services [17] and private bariatric clinics [18]. Indeed, this study found that having an extremely high BMI of >60 kg/m^2^ may negatively impact short-term weight loss success, particularly for non-surgical care.

Consistent with previous studies, the study found that bariatric surgery is more effective than non-surgical care for weight loss [19]. Despite our expectations and previous studies [14, 20], the DACOS scoring system suggests that having type 2 diabetes and non-alcoholic fatty liver disease, which often coexist, may promote weight loss. This might be explained by a highly motivated group of patients in the study population concerned about both having type 2 diabetes and accessing bariatric surgery. Even though type 2 diabetes is an indication of eligibility to access publicly funded bariatric surgery in Australia [21], it is associated with longer wait times in similar healthcare settings [22]. There is also some evidence suggesting that a history of increased glucose levels [23] and willingness to talk to a diabetes educator [24] predict weight loss. Furthermore, weight loss is associated with decreased need for weight gain-promoting medications such as inulin [25], which might promote weight loss in insulin users. However, despite these plausible explanations, the unexpected and contradictory findings might have been due to the small size of the cohort.

The DACOS scoring system suggests that having a history of binge eating disorder can negatively influence weight loss at 6 months and positively influence weight loss at 9 or 12 months. These conflicting findings are somewhat inconsistent with studies showing little or no association between binge eating disorder and weight loss with bariatric surgery [26]. It is possible that the higher rate of lost to follow-up among people with a history of binge eating was due to dissatisfaction with their care or weight loss. A previous study found that participants with higher Binge Eating Scale scores were more likely to withdraw from intensive behavioral weight management treatment (non-surgical care) after the first 6 months of the intervention than those with lower scores [27]. Patients with a history of binge eating disorder who maintain their treatment may be more satisfied with their care, which could lead to greater weight loss. For example, the Look AHEAD trial found that patients with remitted binge eating symptoms lost more weight long-term than those with active binge eating symptoms [28]. A further study found that patients with binge eating disorder who achieved remission with behavioral weight loss treatment lost more weight at 6 and 12 months than those who did not respond [29]. Furthermore, Semaglutide 2.4 mg, a new anti-obesity drug taken once weekly, was shown to be effective in improving short- and long-term control of eating, which was positively associated with substantial weight loss [30]. The episodic nature of binge eating disorder requires clinicians to conduct regular assessments for monitoring and management to maximize the likelihood of successful weight loss outcomes.

The DACOS scoring system suggests that other factors, such as history of depression, anxiety, sleep disorders, high weekly alcohol consumption, heart conditions, and history of osteoarthritis, may negatively affect weight loss at different follow-ups. Depression and anxiety disorders are predictive of less weight loss, particularly after bariatric surgery compared to non-surgical care [31, 32]. There is conflicting evidence on whether common sleep disorders, such as obstructive sleep apnea, predict less weight loss success after bariatric surgery [33, 34]. Regular alcohol consumption is associated with less weight loss success with non-surgical care, according to the Look AHEAD trial [35]. However, it may have a positive effect on weight loss after bariatric surgery, likely because some patients cease or reduce drinking alcohol after surgery [36]. Osteoarthritis is consistently associated with less weight loss after bariatric surgery [37, 38], most likely because of the related musculoskeletal pain limiting physical activity [39]. Although effect sizes were small for ischemic heart disease, the presence and severity of known heart disease may be positively associated with weight loss motivation [40]. This is an encouraging finding given the long-term benefits of weight loss for improving the management of atrial fibrillation [41], which was shown to have a negative impact on weight loss in the DACOS population. Thus, the DACOS scoring system suggests that these factors may vary in importance to some patients.

### Strengths and Limitations

The study’s findings suggest that the DACOS scoring system may be a useful tool for clinicians to consider when discussing potential weight loss predictors with patients after surgery or non-surgical care. However, the study has some limitations. First, the study was conducted in specialized clinical obesity services in a single country, and the results may not be generalizable to other settings. Second, the study’s design and data collection method mean that we cannot establish causality between the predictors and weight loss. Third, the study’s small sample size limits the applicability of the results to all patients. Fourth, data entry errors and incomplete data from patient records could have caused bias. Fifth, it is possible that there are other variables, beyond baseline, that are predictive of weight loss, which were not routinely assessed in practice. Sixth, the DACOS scoring system is likely to underestimate weight loss in the future, as emerging highly effective anti-obesity medications become more widely available in healthcare settings [42]. Notwithstanding these limitations, the study’s findings offer valuable insights into some factors associated with weight loss after bariatric surgery and non-surgical care. These findings have the potential to inform the development of more robust decision-support tools to predict weight loss outcomes within various surgical and non-surgical obesity treatments.

## Conclusion

Overall, the DACOS scoring system is a promising pilot tool for predicting weight loss in patients. The results of this study emphasize the need to consider several factors potentially affecting weight loss in the assessment and management of patients when making treatment decisions. However, to improve the DACOS scoring system’s predictive performance and confirm our findings, further research is needed with a larger, prospective cohort and longer follow-up. The DACOS scoring system should also be validated in other clinical settings, such as primary care and community-based obesity programs.

### Supplementary Information

ESM 1 (DOCX 31 kb)

ESM 2 (DOCX 67 kb)
