# Supplementary material for: Developing a Decision Aid for Clinical Obesity Services in the Real World: the DACOS Nationwide Pilot Study
Source: Obes Surg. 2024 Mar 11;34(6):2073–83. doi: 10.1007/s11695-024-07123-6 (PMC11127827; doi:10.1007/s11695-024-07123-6)
Supplement: Supplementary file 1 — (DOCX 31 kb) [file 11695_2024_7123_MOESM1_ESM.docx]

**Supplementary 1**

**Figure 1: Flow diagram of patients in the DACOS study**

n=273 data records from eight clinics

(number per site: 23, 26, 30, 30, 30, 34, 50, and 50)

Age

- Exclude n=38 aged <16 years

n=235, aged ≥16 years

(number per clinics: 23, 26, 30, 30 ,30, 34, 50, and 12)

Weight recording (a)

- Exclude n=1 missing weight at baseline
- Exclude n=15 missing weight at 6 months

Analysed n=219 at 6 months

- n=204 no bariatric surgery yet
- n=16 received bariatric surgery

Weight recording (b)

- n=66 missing weight at 9 or 12 months

Analysed n=153 at 9 or 12 months

- n=97 no bariatric surgery yet
- n=56 received bariatric surgery surgery

Footnotes

(a) 42/235 records had missing weight at 6 months but n=27 were imputed by linear interpolation

(b) 117/219 records had missing weight at 12 months but n=8 were imputed by linear interpolation and n=43 were replaced by weight at 9 months.
