# Supplementary material for: Developing a Decision Aid for Clinical Obesity Services in the Real World: the DACOS Nationwide Pilot Study
Source: Obes Surg. 2024 Mar 11;34(6):2073–83. doi: 10.1007/s11695-024-07123-6 (PMC11127827; doi:10.1007/s11695-024-07123-6)

**Supplementary 2**

Supplementary analyses: Comparing predicted %TWL at 12 months between DACOS and MBSAQIP

As an external validation, we compared the %TWL predicted by our DACOS tool with the %TWL predictions from the American College of Surgeons Metabolic and Bariatric Surgery Accreditation and Quality Improvement Program (MBSAQIP) surgical risk/benefit calculator.

In our dataset, we had percentage weight loss data at 12 months for 56 patients allocated to surgical treatment. Seven of these patients were 16 or 17 years old at their initial visit and were therefore excluded from the comparison with MBSAQIP.

The comparison between DACOS and MBSAQIP is not entirely consistent:

Starting date: DACOS uses the patient's first visit to an obesity clinic as the starting date, which could be months before surgery. The MBSAQIP, however, uses the date of surgery. This difference may lead to correlation rather than perfect agreement between their predicted weight loss values.

Race and ethnicity: We have differences in how race and ethnicity are coded. We combined "Aboriginal and Torres Strait Islanders" with "American Indian and Alaskan Native" and categorized them as non-Hispanic. All other participants were coded as "White" and non-Hispanic. MBSAQIP uses its default values for these variables.

Missing data: Some variables like "ASA class," "History of PE," and "Steroid Use for Chronic Condition" were missing in our dataset. For these, we left MBSAQIP with its default values ("1. Healthy Patient," "no," and "no").

Diabetes: We had to assume that all patients with diabetes in our dataset did not require insulin or dialysis.

Type of surgery: Our dataset often lacked specific information about the type of surgery performed. Therefore, we compared our predictions to the MBSAQIP predictions for all four main surgery categories.

This information highlights the potential limitations of comparing our results with MBSAQIP due to discrepancies in data and analysis methods. We acknowledge these limitations and interpret the results accordingly.

The DACOS instrument's predictions of %TWL at 12 months have a Mean Absolute Error (MAE) that is on average comparable to or lower than those from the MBSAQIP instrument. This discrepancy may be related to the differences in baseline variables chosen by each instrument.

Interestingly, DACOS and MBSAQIP show similar correlations with observed values, each explaining roughly the same amount of variance in 12-month %TWL. However, both correlations remain relatively low, suggesting neither tool captures all influences on patient outcomes.

Table 1: Mean absolute error and correlations between predicted and observed % weight loss at 12 months

| %TWL at 12 months | MAE | Pearson's correlation, r= |
| --- | --- | --- |
| DACOS predicted vs. observed | 6.9 | 0.334 |
| MBSAQIP predicted (band) vs. observed | 11.5 | 0.251 |
| MBSAQIP predicted (lap sleeve) vs. observed | 6.8 | 0.329 |
| MBSAQIP predicted (lap bypass) vs. Observed | 9.7 | 0.356 |
| MBSAQIP predicted (BPD/DS) vs. Observed | 15.1 | 0.358 |

The low correlations highlight a common problem with predictions: individuals are quite unpredictable. Table 2 further supports this by showing that the standard deviations of observed %TWL are significantly higher than those of predicted weight loss. This finding is also visually evident in Figure 1.

Table 2: Variation in %TWL at 12 months, observed and predicted

|  | Standard deviation |
| --- | --- |
| Observed | 9.24 |
| DACOS predicted | 2.88 |
| MBSAQIP predicted (band) | 4.43 |
| MBSAQIP predicted (lap sleeve) | 3.11 |
| MBSAQIP predicted (lap bypass) | 2.77 |
| MBSAQIP predicted (BPD/DS) | 2.63 |

Figure 1: Distributions of observed weight loss, predicted weight losses from DACOS and predicted weight losses from MBSAQIP


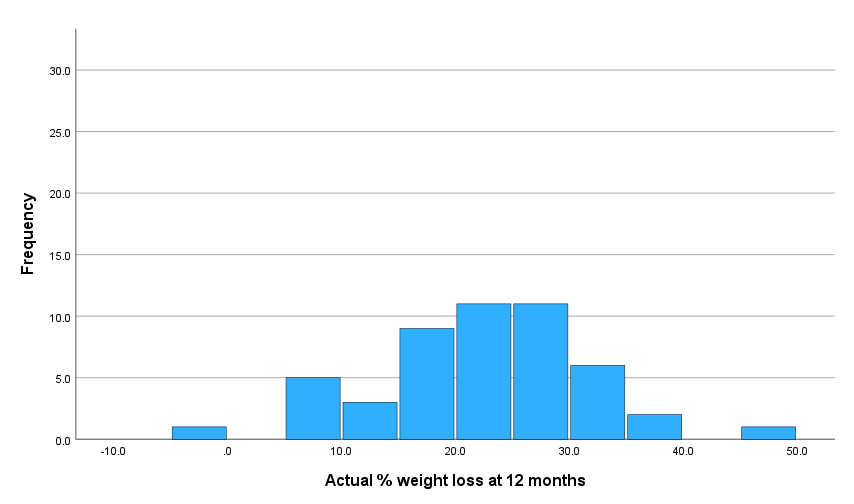

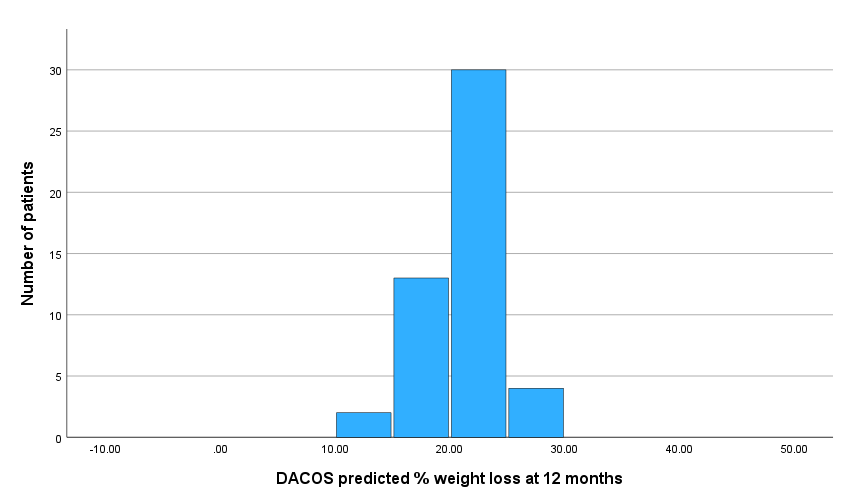


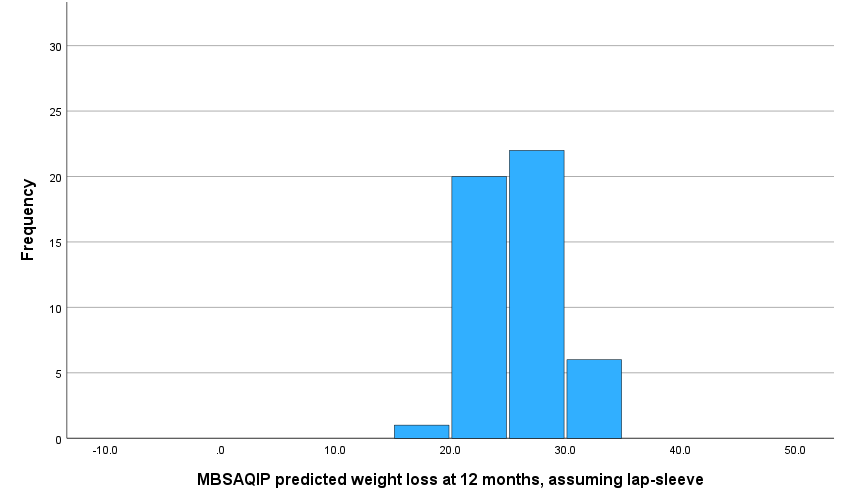

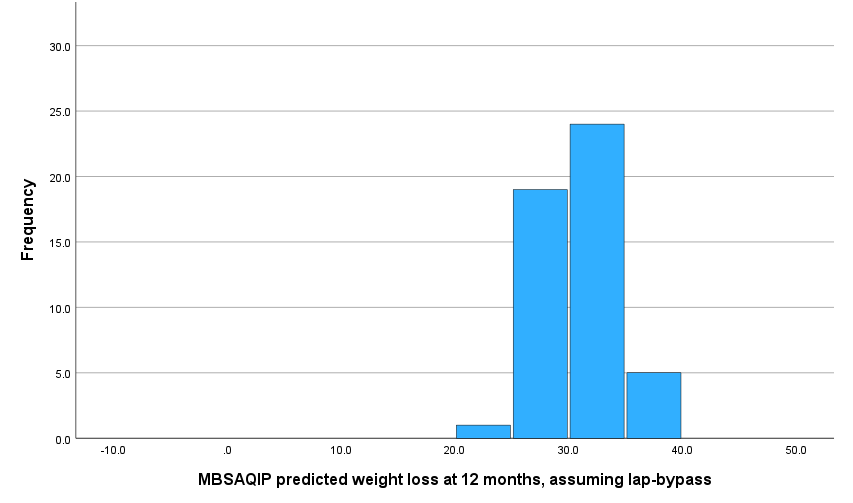

Supplement: Supplementary file 2 — (DOCX 67 kb) [file 11695_2024_7123_MOESM2_ESM.docx]
